# Transcriptional regulation of the virulence genes and the biofilm formation associated operons in *Vibrio parahaemolyticus*

**DOI:** 10.1186/s13099-021-00410-y

**Published:** 2021-03-02

**Authors:** Yiquan Zhang, Yue Qiu, Xingfan Xue, Miaomiao Zhang, Junfang Sun, Xue Li, Lingfei Hu, Zhe Yin, Wenhui Yang, Renfei Lu, Dongsheng Zhou

**Affiliations:** 1 Wuxi School of Medicine, Jiangnan University, Wuxi, 214122 Jiangsu China; 2 School of Medicine, Jiangsu University, Zhenjiang, 212013 Jiangsu China; 3 State Key Laboratory of Pathogen and Biosecurity, Beijing Institute of Microbiology and Epidemiology, Beijing, 100071 China; 4 Department of Clinical Laboratory, Nantong Third Hospital Affiliated to Nantong University, Nantong, 212006 Jiangsu China

**Keywords:** *Vibrio parahaemolyticus*, QsvR, ToxR, CalR, *mfp*

## Abstract

**Background:**

The membrane fusion protein (*mfp*) gene locus of *Vibrio parahaemolyticus* consists of two operons, *cpsQ*-*mfpABC* and *mfpABC*, which are both required for biofilm formation. ToxR and CalR are required for the full virulence of *V. parahaemolyticus*, and their mutual regulation has been demonstrated. Moreover, cell density-dependent expression of *toxR* was previously observed in *V. parahaemolyticus*, but details about the related mechanisms remained unclear. QsvR can work with the master quorum sensing (QS) regulators AphA and OpaR to regulate virulence expression and biofilm formation.

**Results:**

In the present work, we showed that QsvR bound to the promoter-proximal DNA regions of *toxR* and *calR* to repress their transcription as well as occupying the regulatory regions of *cpsQ*-*mfpABC* and *mfpABC* to activate their transcription. Thus, we reconstructed the QsvR-dependent promoter organization of *toxR*, *calR*, *cpsQ*-*mfpABC*, and *mfpABC*.

**Conclusion:**

QsvR directly repressed *toxR* and *calR* transcription as well as directly activated *cpsQ*-*mfpABC* and *mfpABC* transcription. The data presented here promotes us to gain deeper knowledge of the regulatory network of the *mfp* locus in *V. parahaemolyticus*.

## Background

*Vibrio parahaemolyticus*, a Gram-negative halophilic bacterium, naturally inhabits coastal ecosystems and can cause human illness via consumption of raw or undercooked seafood or, less commonly, through small open wounds exposed to seawater [1]. The major clinical symptoms of *V. parahaemolyticus* infection include watery diarrhea, abdominal cramps, nausea, vomiting, chills, and fever [1]. Less frequently, *V. parahaemolyticus* infection may lead to cellulitis (or necrotizing fasciitis) with swelling and pain at the site of infection or septicemia with low blood pressure and shock [1]. The major virulence factors expressed by *V. parahaemolyticus* include thermostable direct hemolysin (TDH), TDH-related hemolysin (TRH), type III secretion system (T3SS) 1 and T3SS2 [1].

The membrane fusion protein (*mfp*) gene locus (VPA1446-1443) consists of two operons *cpsQ*-*mfpABC* and *mfpABC* [2]. *cpsQ* encodes a c-di-GMP binding protein that acts as a positive regulator of capsular polysaccharide (*cps*) genes and *mfpABC* transcription [3]. *mfpA* encodes a potential secreted calcium-binding protein, *mfpB* encodes a potential ABC-type transporter, and *mfpC* encodes a type 1 secretion membrane fusion protein homologous to HlyD [3]. Proteins encoded by the *mfp* locus are required for biofilm formation by *V. parahaemolyticus*, and *mfp* mutants have severe defects in biofilm formation and display altered colony morphology and color when grown on Congo red medium [4]. The master quorum sensing (QS) regulators AphA and OpaR oppositely regulate the transcription of *cpsQ*-*mfpABC* and *mfpABC* during different stages of *V. parahaemolyticus* growth, leading to gradual increases in their transcription with the transition from low cell density (LCD) to high cell density (HCD) [2]. The LysR-type transcriptional regulator CalR is calcium-regulated transcription factor that also can bind to the upstream DNA regions of *mfpABC* to activate its transcription [5, 6].

In addition to directly regulating *mfpABC* transcription, CalR was shown to be involved in directly regulating transcription of the type VI secretion system 2 (T6SS2) gene, *tdh2* and T3SS1 genes as well as swarming motility [5–8]. In addition, transcription of *calR* is directly activated by the transmembrane regulator ToxR, which was first described as a transcriptional activator of cholera toxin [7, 9, 10]. As a feedback of ToxR activation, CalR represses its own transcription and that of *toxR* in a direct manner [7]. Moreover, ToxR binds to the promoter-proximal DNA regions of T3SS1 genes to repress their transcription, and occupies the regulatory regions of *tdh2* and T3SS2 genes to activate their transcription [10–13]. ToxR is also required for biofilm formation, motility, and stress tolerance of *V. parahaemolyticus* [10, 14]. Expression of ToxR itself is dependent on cell density in *V. parahaemolyticus* [11]. However, the master QS regulators AphA and OpaR do not directly regulate *toxR* transcription [11]. Although autorepression of ToxR may contribute to cell density-dependent transcription [11], there are likely other unknown regulators contributing to this process.

The QS and virulence regulator QsvR, an AraC-type transcriptional protein, was originally described as a repressor of biofilm formation in in *V. parahaemolyticus*, as *qsvR* mutant formed robust and distinctive puffball-shaped biofilms [4]. Recently work demonstrated that expression levels of QsvR were consistent with those of OpaR, and both occurred at HCD [15]. In addition, QsvR directly represses *aphA* but activates *opaR* transcription, thereby working with the QS system to tightly regulate the expression of major virulence gene loci such as T3SS1, T3SS2, T6SS2, and *tdh2* [15–17]. In order to detect whether QsvR contributes to cell density-dependent transcription of *toxR*, we performed a series of experiments to investigate the regulatory actions of QsvR on *toxR* transcription. ToxR directly activates the transcription of *calR* [7], whereas CalR directly activates the transcription of *mfpABC* [5]. Therefore, we also detected whether QsvR regulates transcription of both *calR* and the genes within the *mfp* locus. The results showed that transcription of *toxR*, *calR*, *cpsQ*-*mfpABC*, and *mfpABC* were under the direct regulation of QsvR. QsvR represses *toxR* and *calR* transcription as well as activating the transcription of *cpsQ*-*mfpABC* and *mfpABC*.

## Results

### QsvR represses *toxR* transcription

**Fig. 1:**
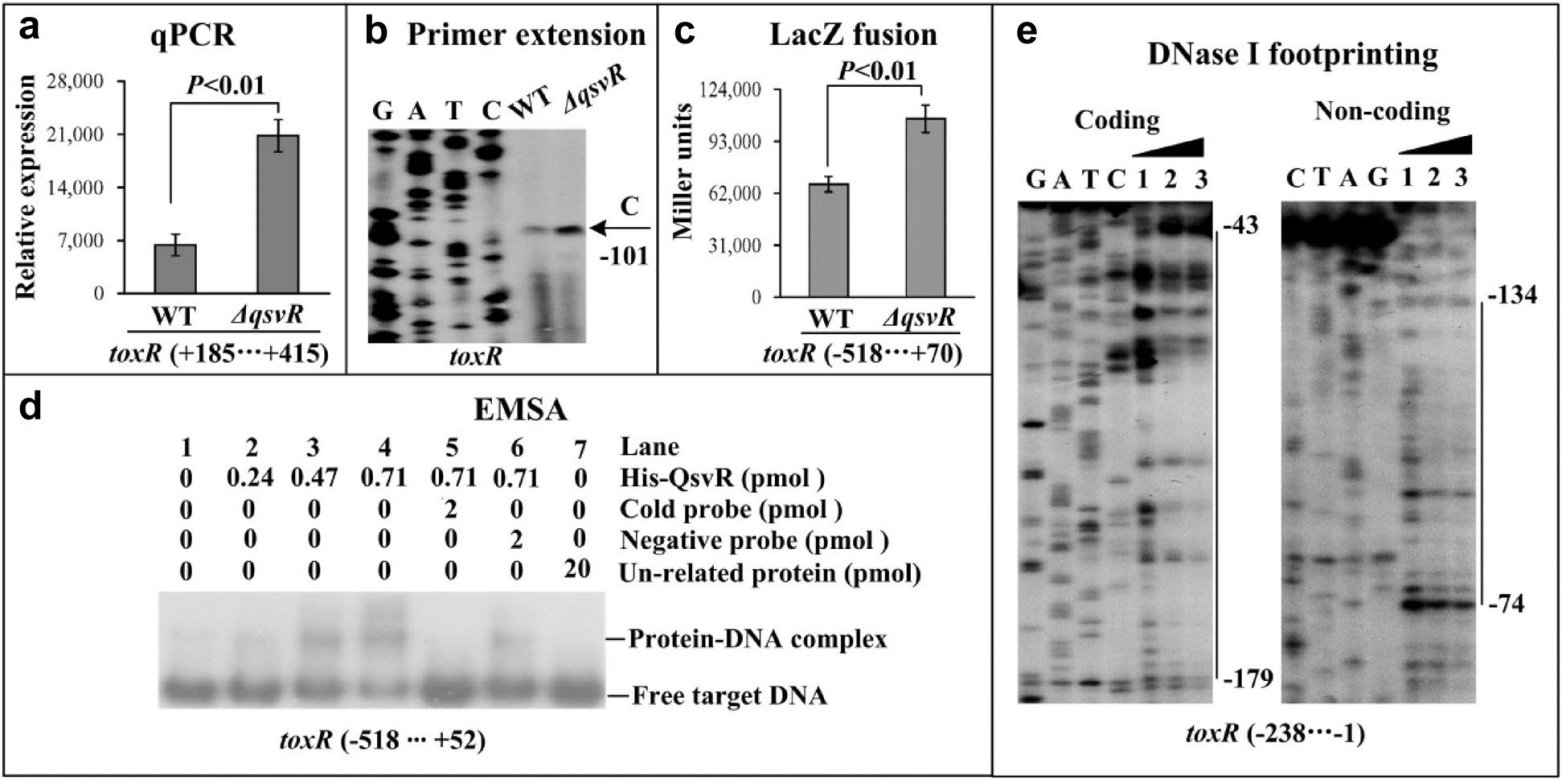
Regulation of *toxR* by QsvR. *V. parahaemolyticus* strains were grown in HI broth at 37 °C, and bacterial cells were harvested at an OD_600_ value of 0.8. Negative and positive numbers indicate the nucleotide positions upstream and downstream of *toxR*, respectively. **a** qPCR. Relative mRNA levels of *toxR* were tested in WT and *ΔqsvR* strains. **b** Primer extension. An oligonucleotide primer complementary to the *toxR* RNA transcript was designed. The primer extension products were analyzed with an 8 M urea-6% acrylamide sequencing gel. **c** LacZ fusion. The promoter-proximal DNA region of *toxR* was cloned into the pHRP309 plasmid and then transferred into WT and *ΔqsvR* strains to determine promoter activity (Miller units) in the cellular extracts. **d** EMSA. The radioactively-labelled promoter-proximal DNA fragments of *toxR* were incubated with increasing amounts of His-QsvR and analyzed using 4% (w/v) polyacrylamide gel electrophoresis. **e** DNase I footprinting. Labelled coding or noncoding DNA probes were incubated with increasing amounts of His-QsvR and analyzed using DNase I footprinting. The footprint regions are indicated by vertical bars at the corresponding sequence positions. Lanes C, T, A and G represent Sanger sequencing reactions

The highest reported levels of *toxR* transcription are seen at optical density at 600 nM (OD_600_) values between 0.2 and 0.4 in *V. parahaemolyticus* strains grown in completed heart infusion (HI) broth at 37 °C [11]. However, the master QS regulators AphA and OpaR do not seem to directly regulate *toxR* transcription [11]. The highest expression levels of QsvR occur at OD_600_ values between 0.4 and 0.8, which is similar to OpaR [15]. Additionally, QsvR directly activates *opaR* transcription [15]. Thus, we decided to assess whether QsvR contributed to cell density-dependent *toxR* transcription. Bacterial cells were harvested at an OD_600_ value of 0.8 and analyzed using quantitative real-tome PCR (qPCR) and primer extension assays (Figs. 1a and b). The results showed that mRNA levels of *toxR* increased significantly in the *ΔqsvR* strain compared with the wild-type (WT) strain, suggesting that *toxR* transcription was negatively regulated by QsvR. The promoter-proximal DNA region of *toxR* was cloned into the plasmid pHRP309, which contains a promoterless *lacZ* reporter gene. The recombinant plasmid was then transferred into the *ΔqsvR* and WT strains, which were analyzed using LacZ fusion assays. The results showed that the promoter activity of *toxR* was much higher in the *ΔqsvR* strain than that in the WT strain, suggesting that the promoter activity of *toxR* was negatively regulated by QsvR (Fig. 1c). The promoter-proximal DNA region of *toxR* was obtained by PCR, and then subjected to electrophoretic mobility shift assay (EMSA). The results showed that His-QsvR was able to specifically bind to the promoter-proximal DNA fragment of *toxR* in a dose-dependent manner in vitro (Fig. 1d). A DNase I footprinting assay was then employed to detect the QsvR binding sites within the upstream DNA fragment of *toxR*. As shown in Fig. 1e, His-QsvR protected a single DNA region from 179 to 43 bp upstream of *toxR* against DNase I digestion. Taken together, these results suggested that QsvR directly repressed the transcription of *toxR*.

### QsvR represses *calR* transcription

**Fig. 2:**
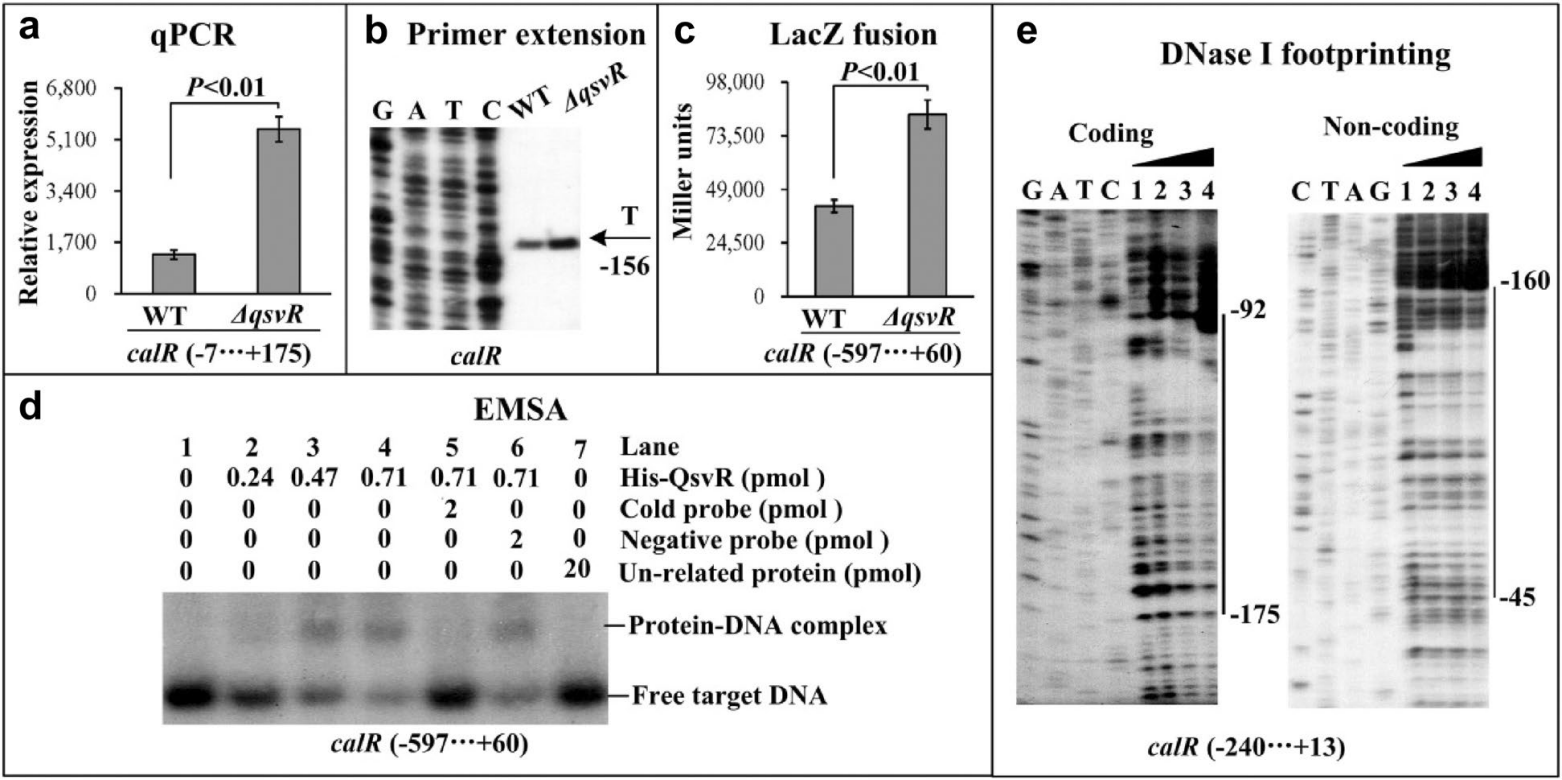
Regulation of *calR* by QsvR. Negative and positive numbers indicate the nucleotide positions upstream and downstream of *calR*, respectively. Lanes C, T, A and G represent Sanger sequencing reactions. The qPCR (**a**), primer extension (**b**), LacZ fusion (**c**), EMSA (**d**) and DNase I footprinting (**e**) were performed as described in Fig. 1

A previous study demonstrated that ToxR specifically bound to the promoter-proximal DNA region of *calR* to activate its transcription [7]. The direct repression of *toxR* transcription by QsvR indicated that the transcription of *calR* might be also regulated by QsvR in *V. parahaemolyticus*. Therefore, we employed qPCR and primer extension assays to test QsvR-dependent transcription of *calR*. The results showed that mRNA levels of *calR* increased significantly in the *ΔqsvR* strain compared to the WT strain, indicating that transcription of *calR* was negatively regulated by QsvR (Figs. 2a and b). The result of LacZ fusion assays showed that the promoter activity of *calR* was significantly enhanced in the *ΔqsvR* strain compared to the WT strain (Fig. 2c). The result of the EMSA demonstrated that His-QsvR was able to specifically bind to the promoter-proximal DNA fragments of *calR* in a dose-dependent manner (Fig. 2d). The DNase I footprinting assay further detected a single His-QsvR binding site located from 175 to 45 bp upstream of *calR*. Thus, the transcription of *calR* was directly repressed by QsvR in *V. parahaemolyticus*.

### QsvR activates the transcription of *cpsQ*-*mfpABC* and *mfpABC*

**Fig. 3:**
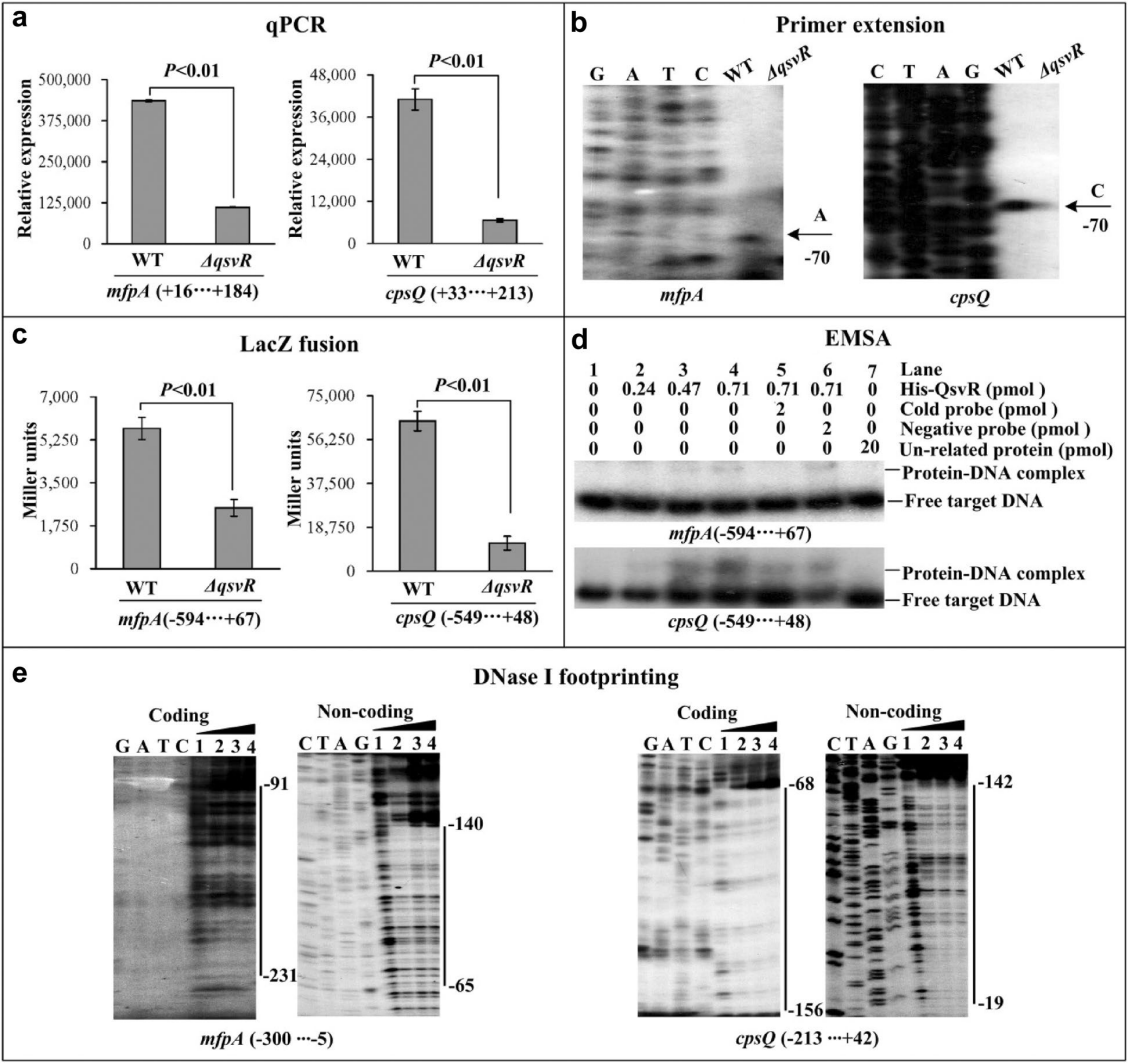
Regulation of *cpsQ*-*mfpABC* and *mfpABC* by QsvR. Negative and positive numbers indicate the nucleotide positions upstream and downstream of *cpsQ*-*mfpABC* and *mfpABC*, respectively. Lanes C, T, A and G represent Sanger sequencing reactions. The qPCR (**a**), primer extension (**b**), LacZ fusion (**c**), EMSA (**d**) and DNase I footprinting (**e**) were performed as described in Fig. 1

CalR directly activates the transcription of *mfpABC* but indirectly activates *cpsQ*-*mfpABC* transcription [5]. Thus, the direct regulation of *calR* by QsvR promoted us to detect whether QsvR has regulatory actions on the transcription of *cpsQ*-*mfpABC* and *mfpABC* in *V. parahaemolyticus*. qPCR (Fig. 3a) and primer extension (Fig. 3b) assays were carried out to measure mRNA levels of *cpsQ* and *mfpA* in *ΔqsvR* and WT strains. The results showed that mRNA levels of both genes were significantly lower in the *ΔqsvR* strain compared to the WT strain. The LacZ fusion assay demonstrated that the promoter activities of *cpsQ* and *mfpA* were also significantly lower in the *ΔqsvR* strain compared to the WT strain (Fig. 3c). In vitro EMSA results showed that His-QsvR was able to specifically bind to the promoter-proximal DNA fragments of both *cpsQ* and *cpsA* in a dose-dependent manner (Fig. 3d). As further determined by DNase I footprinting assay (Fig. 3e), His-QsvR protected a single DNA region for each of the promoter-proximal DNA region of *cpsQ* and *mfpA* against DNase I digestion, located from 156 to 19 bp upstream of *cpsQ* and from 231 to 65 bp upstream of *mfpA*. Taken together, these results demonstrated that QsvR directly activated the transcription of both *cpsQ*-*mfpABC* and *mfpABC* in *V. parahaemolyticus*.

### ToxR exerts no regulatory action on the promoter activities of *cpsQ*-*mfpABC* or *mfpABC*

**Fig. 4:**
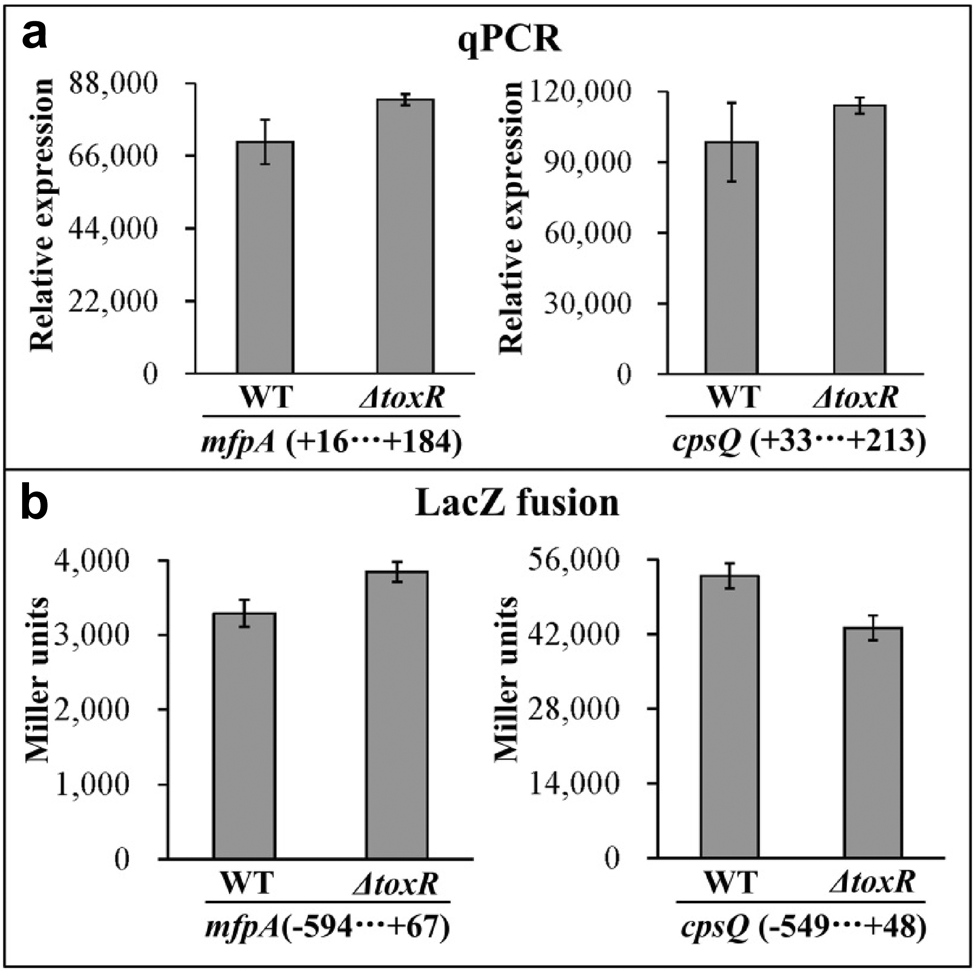
Regulation of *cpsQ*-*mfpABC* and *mfpABC* by ToxR. *V. parahaemolyticus* strains were grown in HI broth at 37 °C, and bacterial cells were harvested at an OD_600_ value of 0.4. The qPCR (**a**) and LacZ fusion (**b**) were performed as described in Fig. 1

Bacterial cells were harvested at an OD_600_ value of 0.4, and then subjected to the qPCR and LacZ fusion assays to investigate ToxR-mediated *cpsQ*-*mfpABC* and *mfpABC* transcription [11]. As shown in Fig. 4, mRNA levels of both *cpsQ* and *mfpA* were similar in *ΔtoxR* and WT strains, and β-galactosidase activity in the cellular extracts of *cpsQ* and *mfpA* were similar to those of the *ΔtoxR* and WT strains. These results suggested that ToxR had no regulatory effect on the transcription of *cpsQ*-*mfpABC* or *mfpABC*.

## Discussion

**Fig. 5:**
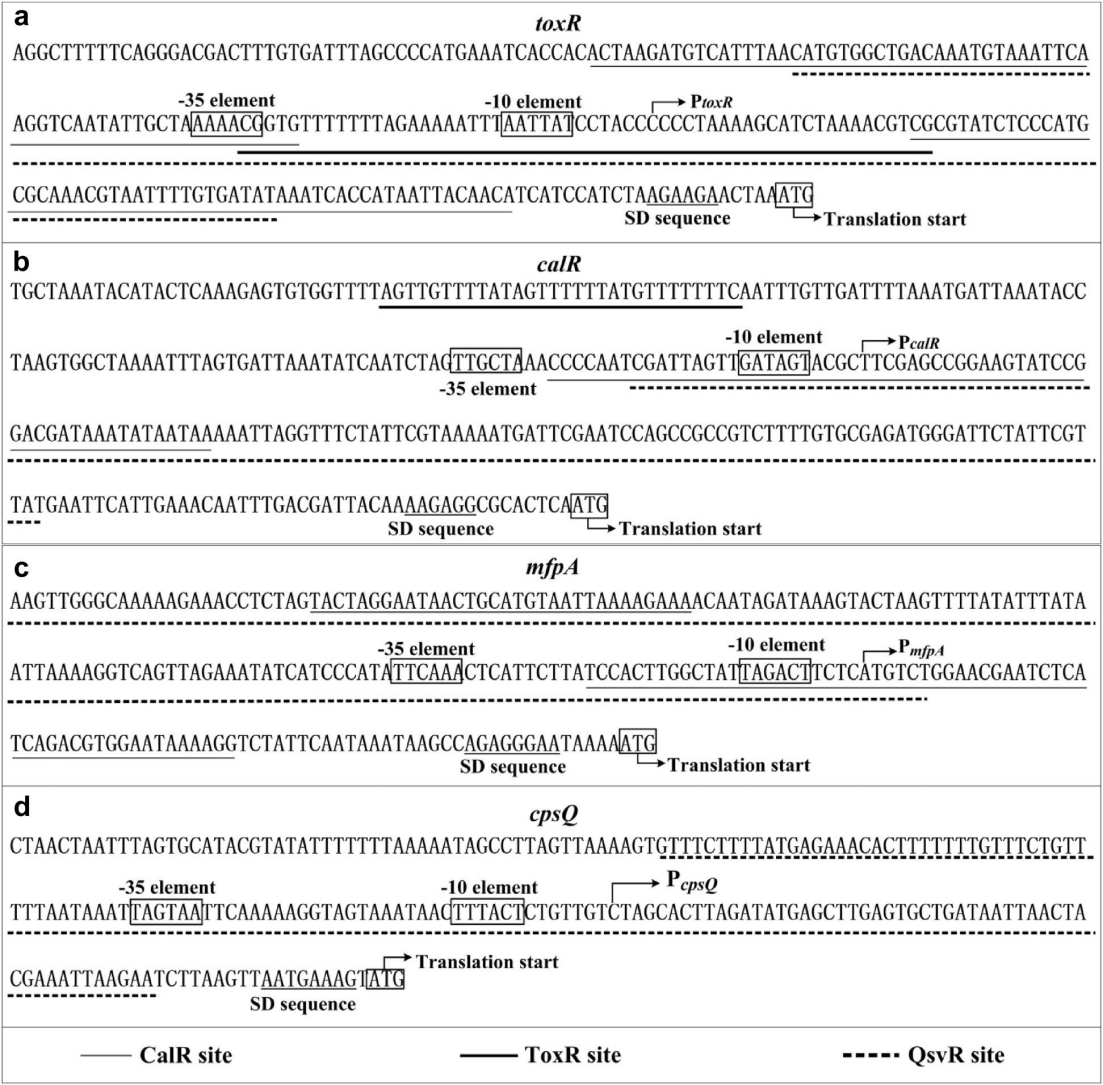
Promoter structure of target genes. The promoter DNA regions of indicated genes were derived from RIMD 2210633. The translation and transcription starts are shown with bent arrows. The predicted core promoter -10 and -35 elements and the SD sequences are boxed. The binding sites of regulators are underlined

High transcriptional levels of *toxR* were observed in *V. parahaemolyticus* when the bacteria was grown in HI broth and harvested at OD_600_ values between 0.2 and 0.4 [11]. However, the reasons for this phenomenon are still not full understood. The data presented here showed that QsvR bound to the promoter-proximal DNA region of *toxR* to repress its transcription in bacterial cells harvested during the mid-logarithmic growth phase. Negative autoregulation of ToxR at LCD (or during the LCD-to-HCD transition) and repression of *toxR* by CalR during the mid-logarithmic growth phase have previously been demonstrated in *V. parahaemolyticus* [7, 11]. Thus, we hypothesized that the cell density-dependent transcription of *toxR* was likely due to the synergistic and sequential regulation of ToxR, CalR and QsvR throughout the growth cycle. At LCD (or during the LCD-to-HCD transition), ToxR binds to its own promoter to repress its own gene transcription via direct interference with the action of RNA polymerase (RNAP) [11]. At HCD, the bacterium replaced ToxR with CalR and QsvR, leading to repression of *toxR* transcription. The binding sites of CalR and QsvR overlap each other as well as the -35 and -10 elements and transcription start site of *toxR* (Fig. 5a). Thus, QsvR may work with CalR to silence the transcription of *toxR* by directly interfering with RNAP action.

Direct activation of *calR* by ToxR and feedback repression of *toxR* by CalR have previously been reported [7]. The direct repression of *toxR* by QsvR reported in the present study promoted us to detect whether QsvR regulated *calR* transcription. The data showed that QsvR could bind to the promoter-proximal DNA region of *calR* to repress its transcription. The binding site of QsvR for *calR* overlaps with the CalR site as well as the -35 and -10 elements and transcription start site of *calR* (Fig. 5b). Thus, QsvR may also work with CalR to silence the transcription of *calR* by directly interfering with RNAP action. ToxR activation of *calR* transcription belongs to class I transcriptional stimulation, as its binding site is far from the -35 element [7, 18]. Direct regulation of *calR* and *toxR* transcription by QsvR indicated that QsvR controlled all of the genes within the CalR and ToxR regulons in *V. parahaemolyticus*.

**Fig. 6:**
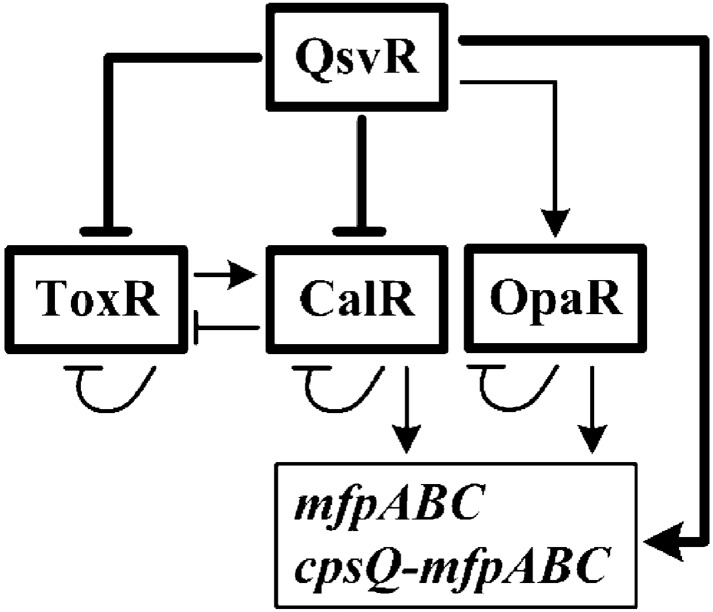
Regulatory circuit. Arrows represent positive regulation, and vertical lines represent negative regulation. Elements described in previous studies are indicated with thin lines, and those described in the current study are indicated with bold lines

The data presented here also showed that QsvR bound to the promoter-proximal DNA regions of *cpsQ*-*mfpABC* and *mfpABC* to activate their transcription. The binding sites of QsvR for *cpsQ*-*mfpABC* and *mfpABC* also overlap with their −35 and −10 elements and transcription start sites (Figs. 5c and d). This is an abnormal mechanism for a transcriptional regulator to activate transcription of its target genes. However, this phenomenon is expected because similar QsvR-dependent promoters have been found in *V. parahaemolyticus* [15]. There are likely additional unknown regulators that repress the transcription of *mfp* genes in *V. parahaemolyticus* at the same growth conditions. QsvR may antagonizer these repressors, leading to the activation of transcription of genes within the *mfp* locus [8]. Both CalR and OpaR have been reported to be required for the expression of the *mfp* locus [2, 5], whereas transcription of *calR* and *opaR* is under the direct control of QsvR according to data presented here and in the Ref. [15]. Because QsvR directly activates *opaR* transciption, deletion of *qsvR* should also result in reduced expression of OpaR, which may further reduce the expression of genes within the *mfp* locus. The data presented here and in previous studies led us to suggest a complex regulatory circuit involving co-regulation of the *mfp* locus by QsvR, OpaR, CalR, and ToxR (Fig. 6), which contributed to a deeper understanding of the regulatory network of the *mfp* locus in *V. parahaemolyticus*. Although the detailed molecular mechanisms are still unclear, both CpsQ and MfpABC have been reported to be required for biofilm formation in *V. parahaemolyticus* [3, 4]. The regulation of *cpsQ*-*mfpABC* and *mfpABC* transcription by QsvR, OpaR and ToxR may be one of the mechanisms regulating biofilm formation in *V. parahaemolyticus*.

## Conclusion

QsvR directly repressed the transcription of *toxR* and *calR*, whereas it directly activated *cpsQ*-*mfpABC* and *mfpABC* transcription. Our results on the regulation of *toxR*, *calR*, *cpsQ*-*mfpABC* and *mfpABC* by QsvR provided us with deeper understanding of the regulatory network of the *mfp* locus in *V. parahaemolyticus*.

## Materials and methods

### Bacterial strains

*V. parahaemolyticus* strain RIMD2210633 (wild type, WT) was used in the current study [19]. Nonpolar *qsvR* and *toxR* single-gene deletion mutants (*ΔqsvR* and *ΔtoxR*) derived from the WT strain were described in our previous studies [11, 15]. All primers used in this study are listed in Table 1.

**Table 1:** Oligonucleotide primers used in this study

Target	Primers (forward/reverse, 5′-3′)
Construction of mutants	
*qsvR*	GTGACTGCAGATGCTAAAAGCGGTGATTC/GATTCAAATGCGATTTCTGTTGGCTGGTGGACGACTAATG
	CATTAGTCGTCCACCAGCCAACAGAAATCGCATTTGAATC/GTGAGCATGCGAGAAGTCTGTAAACGAAACG
	GTGACTGCAGATGCTAAAAGCGGTGATTC/GTGAGCATGCGAGAAGTCTGTAAACGAAACG
*toxR*	GTGACTGCAGAAACGCAATTTGTCTGATG/ATCTTCATGCTGGCCTCCTTTAGTTCTTCTTAGATGGATGATG
	CATCATCCATCTAAGAAGAACTAAAGGAGGCCAGCATGAAGAT/GTGAGCATGCAATTCGGCGGCTTTGTTC
	GTGACTGCAGAAACGCAATTTGTCTGATG/GTGAGCATGCAATTCGGCGGCTTTGTTC
Protein expression	
*qsvR*	AGCGGGATCCATGCCGAACATTGAGATCATTC/AGCGAAGCTTTTAACCTCTTACTACCTGATTACG
qPCR	
*toxR*	TTGTTTGGCGTGAGCAAGG/TAGCAGAGGCGTCATTGTTATC
*calR*	ATGTAAAAAGAAAACCGTACA/AACACAGCAGAATGACCGTG
*cpsQ*	GCCTGAAATCCTAATGCTC/AGTGTCAGAAGGTGTATCAAC
*mfpA*	GCGGGCAATGATCGTCTAAC/TCACCTGAACCTGCGACAAG
Primer extension	
*toxR*	/TTAGTTCTTCTTAGATGGATGATG
*calR*	/GCAAAATATCGGTACTTCA
*cpsQ*	/GATTTCAGGCTTTTCCGTGTAC
*mfpA*	/ATTCCCTCTGGCTTATTTATTG
LacZ fusion	
*toxR*	GCGCGTCGACATCGTTAAGGTATTTGCA/GCGCGAATTCCGAGCGAATTACTATTTGG
*calR*	GCGGTCGACGTTTGTTTGCTCGGATTGTTTG/GCGTCTAGACAAAGTGCTTTCCATACGGTAG
*cpsQ*	GCGCGTCGACCAGACGGGCATTGATAAG/GCGCGAATTCCATTAGGATTTCAGGCTTTT
*mfpA*	GCGCGTCGACTTATGACTTAGATACCGAA/GCGCGAATTCCGAAATCAGCGATATTGTTG
EMSA	
*toxR*	ATCGTTAAGGTATTTGCA/CGAGCGAATTACTATTTGG
*calR*	GTTTGTTTGCTCGGATTGTTTG/CAAAGTGCTTTCCATACGGTAG
*cpsQ*	GTTCCAGCAATACTGACTAAGC/GATTTCAGGCTTTTCCGTGTAC
*mfpA*	TAGGACGCAAGCCACAAG/CGAAATCAGCGATATTGTTG
DNase I footprinting	
*toxR*	TTTCAGGGACGACTTTGTG/TTAGTTCTTCTTAGATGGATGATG
*calR*	ATTCCCTCTGGCTTATTTATTG/CCACGGCATTACTTACTG
*cpsQ*	TACCTAACTAATTTAGTGCA/GATTTCAGGCTTTTCCGTGTAC
*mfpA*	ACATACTATTAAATCGCATC/ATTCCCTCTGGCTTATTTATTG

### Bacterial growth conditions

*Vibrio parahaemolyticus* strains were grown in 2.5% Bacto heart infusion (HI; BD Bioscience, USA) broth at 37 °C with shaking at 250 r/min [15]. Briefly, overnight bacterial cultures were diluted 50-fold into 15 ml of fresh HI broth, and were allowed to grow at 37 °C to OD_600_ ≈ 1.0 (mid-exponential growth phase). Thereafter, the cultures were diluted 1000-fold into 15 ml of fresh HI broth for a third round of cultivation. Bacterial cells were harvested at the required cell densities. When necessary, the medium was supplemented with 50 μg/ml gentamicin.

### Quantitative real-time PCR (qPCR)

The qPCR assay was performed as previously described [20]. Briefly, total RNAs were extracted from *V. parahaemolyticus* strains using the TRIzol Reagent (Invitrogen, USA). Contaminated DNA in the total RNAs was removed using an Ambion’s DNA-free™ Kit (Ambion Inc., USA) according to the manufacturer’s instructions. cDNA was generated using 8 µg of total RNAs and 3 µg of random hexamer primers. The relative mRNA levels of each target gene were determined based on a standard curve of 16S rRNA (reference gene) expression performed for each RNA preparation. The annealing condition for all primer pairs was 54 °C for 4 s.

### Primer extension assay

Primer extension assay was performed as previously described [20, 21]. Briefly, approximately 10 µg of total RNAs were annealed with 1 pmol of 5′- ^32^P-end labelled reverse primer to generate cDNAs using a Primer Extension System (Promega, USA). The same labelled primer was also used for sequencing with the AccuPower & Top DNA Sequencing Kit (Bioneer, Korea) according to the manufacturer’s instructions. The products of primer extension and sequencing were concentrated and analyzed in an 8 M urea-6% polyacrylamide gel electrophoresis, and the results were detected by autoradiography using Fuji Medical X-ray film (Fuji Photo Film Co., Ltd. Japan).

### LacZ fusion and β-galactosidase assay

The promoter-proximal DNA region of each target gene was cloned into the corresponding restriction endonuclease sites of pHRP309, which harbors a promoterless *lacZ* reporter gene and a gentamicin resistance gene [22]. The recombinant plasmid was subsequently transferred into the WT strain and the deletion mutants to measure the β-galactosidase activities of the cellular extracts using a β-Galactosidase Enzyme Assay System (Promega, USA) according to the manufacturer’s instructions [15, 20]. Briefly, the assay was performed by adding 30 μL of diluted sample to an equal volume of assay 2 × buffer that containing the substrate *o*-nitrophenyl-β-d-galactopyranoside. Samples were incubated for approximately 30 min, during which time the β-galactosidase hydrolyzes the colorless substrate to *o*-nitrophenol, which is yellow. The reaction was terminated by the addition of 90 μL sodium carbonate, and the absorbance was read at OD_420_ and OD_550_ with a spectrophotometer. The number of Miller units (representing the galactosidase activity) was calculated using the following formula: 10^6^ × [(OD_420_ − 1.75 × OD_550_)/(T × V × OD_600_)]. The Miller units represent the change in the OD_420_/min/ml relative to the OD_600_ of the cells.

### Preparation of 6× His-tagged QsvR (His-QsvR)

The entire coding region of *qsvR* was cloned into the corresponding restriction endonuclease sites of pET28a plasmid (Novagen, USA). Thereafter, the recombinant plasmid encoding His-QsvR was then transferred into *E. coli* BL21λDE3 for protein expression [23]. Expression and purification of His-QsvR was performed in a manner similar to that described previously for His-OpaR [21].

### Electrophoretic mobility shift assay (EMSA)

EMSA was carried out as previously described [15, 21]. Briefly, the 5′-ends of the promoter-proximal DNA region of each target gene were labelled with [γ-^32^P] ATP using the T4 polynucleotide kinase. EMSA was performed in a 10 µl reaction volume containing binding buffer (1 mM MgCl_2_, 0.5 mM EDTA, 0.5 mM DTT, 50 mM NaCl, 10 mM Tris–HCl/pH 7.5 and 10 mg/ml salmon sperm DNA), labelled DNA probe, and increasing amounts of His-QsvR. Three controls were included in each EMSA experiment: (1) cold probe as a specific DNA competitor (the same unlabeled DNA fragments), (2) negative probe as a non-specific DNA competitor (the unlabeled coding region of the 16S rRNA gene) and (3) non-specific protein competitor (rabbit anti-F1-protein polyclonal antibodies). The products were analyzed in a native 4% (w/v) polyacrylamide gel, and the results were detected by autoradiography after exposure to Fuji Medical X-ray film.

### DNase I footprinting

The DNase I footprinting assay was performed as previously described [15, 21]. Briefly, DNA binding was carried out in 10 µl reaction containing binding buffer, single strand 5′-ends ^32^P-labelled probe, and increasing amounts of His-QsvR and incubated at room temperature for 30 min. Before digestion, 10 µl of Ca^2+^/Mg^2+^ solution (5 mM CaCl_2_ and 10 mM MgCl_2_) was added to each reaction and incubated at at room temperature for 1 min. Optimized RQ1 RNase-Free DNase I (Promega, USA) was added to each reaction mixture, and the mixture was incubated at room temperature for 40-90 s. The reaction was quenched by adding 9 µl of stop solution (200 mM NaCl, 30 mM EDTA, and 1% SDS). The partially digested DNA samples were extracted with phenol/chloroform, precipitated with ethanol, and analyzed in 6% polyacrylamide/8 M urea gels. Protected regions were identified by comparison with sequence ladders. The templates used for DNA sequencing were the same as the DNA fragments used in the DNase I footprinting assays. The results were detected by autoradiography after exposure to Fuji Medical X-ray film.

### Experimental replicates and statistical methods

At least three independent replicates of the LacZ fusion and qPCR assays were performed, and primer extension, EMSA, and DNase I footprinting assays were each performed at least twice. Values are expressed as mean ± standard deviation (SD). Paired Student’s *t*-tests were used to calculate statistically significant differences, and *P* values < 0.01 were considered statistically significant.

## Data Availability

Not applicable.
